# Mechanisms Underlying Hazardous Alcohol Use After Mild Traumatic Brain Injury

**DOI:** 10.35946/arcr.v45.1.09

**Published:** 2025-09-03

**Authors:** Makenzie Patarino, Jenna Sanders, Abigail G. Schindler

**Affiliations:** 1 Department of Psychiatry and Behavioral Sciences, University of Washington, Seattle, Washington; 2 Graduate Program in Neuroscience, University of Washington, Seattle, Washington; 3 Center for Neurobiology of Addiction, Pain & Emotion, University of Washington, Seattle, Washington; 4 VA Northwest Geriatric Research, Education and Clinical Center, VA Puget Sound Health Care System, Seattle, Washington; 5 VA Northwest Mental Illness Research, Education and Clinical Center, VA Puget Sound Health Care System, Seattle, Washington; 6 Department of Medicine, University of Washington, Seattle, Washington

**Keywords:** alcohol, traumatic brain injury, trauma, neuroinflammation, neurodegeneration, dopamine, reward processing, executive function

## Abstract

**PURPOSE:**

Alcohol use disorder (AUD) and mild traumatic brain injury (mTBI) have a bidirectional, synergistic, and complicated relationship. Although it is difficult to definitively say that mTBI causes AUD, certain biological mechanisms that occur after trauma are also associated with hazardous alcohol use. Hazardous drinking is defined as any quantity or pattern of alcohol consumption that places people at risk for physical and/or psychological harm. This review explores how the physiological, emotional, and behavioral consequences of mTBI may lead to worse outcomes after hazardous alcohol use and increase the risk for AUD. AUD is one of the most common comorbid conditions that occurs after mTBI, and thus a clear understanding of the mechanistic changes that influence its onset may help to identify preventative and therapeutic measures for individuals who are at risk. This review provides an overview of recently published studies (from 2021 to 2024) and how these new findings fit into the existing literature.

**SEARCH METHODS:**

This review was conducted by searching “alcohol, traumatic brain injury, TBI” in PubMed, Google Scholar, and Medline databases in October and December 2024. Only articles in English were reviewed. Titles, abstracts, and methods of all articles were read to determine relevance, then the full texts of articles that met inclusion criteria were obtained. The search included articles published after March 2021; relevant papers published before 2021 were identified by consulting previously published reviews on this topic. Articles were excluded if they only discussed (1) moderate/severe TBI, (2) adolescent populations or TBI during adolescence, (3) populations with a history of AUD before TBI, (4) acute outcomes after TBI (less than 2 weeks), or (5) prevalence or effects of TBI while intoxicated. Also excluded were papers that did not specify if TBI preceded or followed hazardous alcohol use or did not discuss the relationship between TBI and alcohol use.

**SEARCH RESULTS:**

The search resulted in 196 articles for initial examination. Of those, 155 were excluded and 42 were included. Eight review papers about alcohol use after TBI published from 2009 to 2023 were also examined, which provided foundational and additional background information on publications from 1990 to 2021.

**DISCUSSION AND CONCLUSIONS:**

This review discusses mechanisms that contribute to negative outcomes after mTBI and hazardous alcohol use and to the development of AUD after mTBI. These include inflammation and immune signaling, neuroendocrine alterations, oxidative stress, neurodegeneration, dopamine signaling, and behavioral impairments. Although current literature on the role of the gut-microbiome axis in this context is limited, this topic is also explored.

There has been significant research on the biological changes that occur after mTBI and on which mechanisms may precede development of AUD; however, few studies have directly measured the outcomes of alcohol use after mTBI in the same experiment. Future preclinical and clinical research that concurrently studies alcohol use and mTBI could help establish causality for the complex relationship between trauma and alcohol use. Improved knowledge could help identify preventative measures and treatment options to improve quality of life for individuals who experience mTBI and hazardous alcohol use.

Traumatic brain injury (TBI) and alcohol use disorder (AUD) are both public health issues, and their high co-occurrence is a pressing concern for the affected individuals, researchers, clinicians, and policymakers.^1^ Approximately one-third of individuals who have history of TBI are diagnosed with AUD after injury, and 33% to 75% of individuals with AUD have a history of TBI.^2^–^5^ TBIs are classified as “mild,” “moderate,” and “severe,” which corresponds to scores of 13 to 15, 9 to 12, and 3 to 8, respectively, on the Glasgow Coma Scale. Mild TBI (mTBI) can also be called a concussion, and the terms are often used interchangeably. mTBI has been estimated to represent nearly 75% of all TBI diagnoses.^3^,^6^ For people who regularly consume alcohol, consumption sharply declines in the month following a TBI diagnosis across all injury severities. Individuals with mTBI tend to resume regular alcohol consumption sooner after injury (generally within 6 to 12 months) and reach moderate to heavy levels of alcohol consumption sooner than individuals with moderate to severe TBI.^7^ (The Dietary Guidelines for Americans 2020–2025 defines moderate drinking as two drinks or less per day for men and one drink or less per day for women, and the National Institute on Alcohol Abuse and Alcoholism (NIAAA) defines heavy drinking as five or more drinks per day or 15 or more drinks per week for males and four drinks or more per day or eight or more drinks per week for females.^8^) This circumstance may be in part because individuals who are diagnosed with mTBI are unlikely to spend time under medical care, whereas people with moderate and severe TBI often have prolonged hospital stays.

Because there is a lack of publications regarding how hazardous alcohol use influences outcomes after moderate to severe TBI, the focus of this review will be on mTBI—although additional research on moderate to severe TBI will improve overall understanding of neurological and peripheral interactions with alcohol. Hazardous drinking is defined as any quantity or pattern of alcohol consumption that places a person at risk for physical and/or psychological harm, which includes (but is not limited to) all-cause mortality, stroke, cancer, cardiovascular disease, hypertension, traumatic injuries, adverse alcohol-drug interactions, and impaired social and occupational functioning.^9^ Hazardous drinking encompasses the NIAAA definitions of either heavy drinking or binge drinking (five or more drinks for males and four or more drinks for females in a period of 2 hours), or a score of eight or more on the Alcohol Use Disorders Identification Test (AUDIT).^10^ Any subsequent descriptions of “hazardous alcohol use” will align with this definition, including the criteria for binge or heavy drinking, unless otherwise stated.

Despite the label of “mild,” mTBI can result in behavioral and physiological symptoms that develop acutely and persist for years after the initial injury.^11^–^16^ Further, individuals with AUD and a history of mTBI generally have worse outcomes, such as more severe symptoms, decreased quality of life, and less responsiveness to treatment.^17^–^19^ Due to the high rate of comorbid AUD and mTBI in the general population and the associated costs to individuals, their loved ones, and society, research on this topic is important to identify opportunities for intervention.

This review does not attempt to answer the question whether mTBI results in the development of AUD (see Olsen and Corrigan^20^ for a discussion on this topic), but rather discusses the mechanistic changes that occur with hazardous drinking following an mTBI that may worsen chronic outcomes and leave an individual susceptible to developing AUD. The review covers six broad categories of biological mechanisms (inflammation and immune signaling, oxidative stress, local cell death and loss of proteostasis, global loss of white and gray matter volume, neuroendocrine alterations, and dopamine signaling) and various behaviors related to executive functioning (cognitive and reward processing deficits, disinhibition, impulsivity, and affect).

Various subsets of the population that experience mTBI and AUD at higher rates are discussed, and only adult populations (age 18 and older) are included (the strong relationship between AUD and prior experience of TBI in adolescence has been previously reviewed).^21^,^22^ Additionally, because this review discusses chronic outcomes after mTBI and mechanisms that influence the onset of AUD, papers about individuals with preexisting AUD and/or TBIs that occurred while intoxicated are not included. It is beyond the purview of this review to compare the biomechanical forces and pathological progression of different types of injury modalities leading to mTBI (e.g., blunt impact, penetrating, blast, or rotational),^23^ but it is important to note that blast exposure is a whole-body injury and often results in polytrauma as a primary diagnosis rather than only mTBI. Like other modalities, blast TBI can result from one acute exposure; however, recent attention also has been focused on understanding how blast TBI can occur in response to multiple subconcussive low-level blasts from the use of high-powered artillery during military training. Peripheral impacts of traumatic injuries are especially relevant when discussing comorbid alcohol use, because alcohol affects multiple systems throughout the body. Although focal mTBIs also affect the body beyond the brain, it may be prudent to specifically explore blast-induced mTBI and alcohol interactions in the future because of the diffuse nature of the injury.

This review provides an overview of studies published from March 2021 to December 2024 and how these new findings fit into the existing literature. Limitations of the surveyed papers and the interpretations in this review are discussed, as well as gaps in the literature and future research avenues of interest for improving outcomes.

## Search Methods

This review was conducted by searching “alcohol, traumatic brain injury, TBI” in PubMed, Google Scholar, and Medline databases. Database searches took place during October and December 2024, with the last search on December 16, 2024. No unpublished results or preprints were included. Only articles in English were reviewed, including articles from five continents (North America, Europe, Asia, Africa, and Australia). Titles, abstracts, and methods of all articles were read to determine relevance, then the full texts of articles that met the inclusion criteria were obtained. The search included articles published after March 2021, and previously published reviews on this topic were consulted to identify relevant papers published before 2021. Articles were excluded if they only discussed (1) moderate/severe TBI (9 articles), (2) adolescent populations or TBI during adolescence (11 articles), (3) populations with a history of AUD before TBI (14 articles), (4) acute outcomes that occurred within 2 weeks after TBI (seven articles), or (5) prevalence or effects of TBI incurred while intoxicated (62 articles). Also excluded were papers that did not specify if TBI preceded or followed hazardous alcohol use (five articles) or that did not discuss the relationship between TBI and alcohol use (46 articles).

## Results of the Literature Search

The search identified 196 articles for initial examination. Of those, 154 were excluded for the reasons described above and 42 were included in the review. Six of the papers described preclinical research, 34 covered human data, and two included both preclinical and clinical results. Eight additional review papers about alcohol use after TBI published between 2009 and 2023 were reviewed, which provided foundational and further background information for publications from 1990 to 2021.

## Results of the Reviewed Studies

### Rates of AUD and Psychiatric Disorders After mTBI

Patterns of alcohol use after mTBI varied based on a number of demographic factors. Younger age, not being in a relationship, previous alcohol use, and intoxication at the time of injury were associated with higher rates of hazardous alcohol use post injury.^24^,^25^ Military personnel and contact-sport athletes experience TBIs and alcohol use at higher levels than the general population; therefore, extra attention was given to these populations in this review.^7^ Of the reviewed studies published between 2021 and 2024 that investigated the relationship between TBI and subsequent alcohol use patterns, eight focused on military populations (service members and veterans), six on contact-sport athletes, and nine on the general population.

Historically, research has found that hazardous alcohol use is more common in military populations with a history of mTBI compared to those in the general population with an mTBI diagnosis.^26^ Previous research has also shown that hazardous alcohol use is more common among veterans with mTBI than those without mTBI, and the rate is even higher for those with comorbid mTBI and post-traumatic stress disorder (PTSD).^27^ Recent findings published between 2021 and 2024 about alcohol use after mTBI, however, were mixed. A handful of studies found similar rates of alcohol consumption and AUD between veterans with and without mTBI, although PTSD, depression, and poor cognitive function were still observed after mTBI.^28^–^30^ In contrast, multiple papers confirmed previous findings of higher levels of alcohol consumption in veterans with mTBI compared to those without.^31^–^36^ Brenner et al. found that service members with a history of mTBI had a 22% higher increase in AUD from preinjury levels compared to those without mTBI.^32^ Steffen-Allen et al. observed a peak in alcohol consumption at 2 years after mTBI at 4.8 drinks per week on average (compared to 2.3 drinks per week preinjury), before dropping to 3.5 drinks per week at 2 to 5 years after mTBI.^36^ The probability of binge drinking (i.e., consuming five or more drinks for men, four or more for women, on any one occasion) increased from 14% preinjury to approximately 30% for the 1 to 5 years after mTBI.

Contact-sport athletes (football, boxing, hockey, soccer, rugby) are also at higher risk for mTBI than the general population.^37^ Because of the length of their participation in sport, the risk for repetitive mTBI and cumulative effects of subconcussive head impacts are a concern specifically for collegiate and professional athletes.^38^ There is also a pervasive culture of alcohol and substance use in many sports, which exposes individuals to hazardous drinking starting in high school and potentially continuing through retirement.^39^ These two factors, along with recent attention to the long-term psychological and cognitive health of contact-sport athletes, have motivated research regarding the association between mTBI and alcohol use in current and former athletes.^39^ Recent findings regarding this association in contact-sport athletes have been mixed. Three papers surveying retired professional football, hockey, and rugby players, respectively, did not find a significant association between number of concussions and alcohol use.^37^–^39^ The results differed for collegiate athletes: not only was mTBI history predictive of alcohol use, but alcohol use in the 4 weeks after sustaining a concussion prolonged recovery time.^17^,^40^ A study of former collegiate football players did not specifically report previous TBI history but determined a higher prevalence of cognitive impairment, headaches, and alcohol use compared to the general male population.^41^ It is possible that these opposing results between retired professional athletes and collegiate athletes is reflective of the role that age plays in influencing outcomes after mTBI and with alcohol use.

The articles identified in the literature search covered a range of subpopulations, including different nationalities (e.g., individuals from Canada, Switzerland, China, and the United States); specific racial or ethnic groups, such as Hispanics; and rural versus urban populations. Although nuances across the recently published studies existed, there was general agreement that alcohol consumption increased after mTBI.^42^,^43^ Multiple studies also found significant increases in the rates of other negative outcomes after mTBI in addition to alcohol use, including depression, aggression-hostility, risk of developing dementia, and poor general mental health.^44^–^48^

Because of the heterogeneity in how mTBI manifests from person to person, one cannot say that mTBI exposure increases hazardous alcohol use for every individual. However, many previous and current studies have supported this association, and for a subset of individuals, mTBI symptoms clearly linger for years after the initial injury. Determining the biological changes that occur after mTBI and that interact with hazardous drinking to worsen outcomes and contribute to the development of AUD will be critical in understanding this complex relationship and identifying effective interventions.

### Biological Mechanisms Connecting TBI and AUD

Multiple distinct mechanistic changes occur after mTBI that are also associated with hazardous drinking. However, although clinical studies can measure biological and behavioral changes and their association with alcohol use and mTBI, it is difficult to prove causality and the degree of contribution in human subjects. Preclinical studies allow for deeper interrogation and play a significant role in understanding how biological outcomes may mediate the connection between mTBI and AUD. Translationally relevant animal models include the fluid-percussion and closed-head weight-drop models for impact TBI, the CHIMERA model for rotational TBI, and blast exposures for blast TBI. The following sections discuss various mechanisms in the context of preclinical and clinical work. The six mechanisms of interest broadly defined here are not exhaustive but are based on the results of the literature search. They represent a holistic view of the plausible substrates that influence chronic outcomes and post-injury alcohol consumption. An in-depth explanation of each mechanism is outside the scope of this review, and readers are referred to the referenced papers for more detailed information on each topic.

#### Inflammation and immune signaling

Both mTBI and alcohol use can lead to increased inflammation and immune signaling. Immune cascades include the release of damage-associated molecular patterns (DAMPs); activation of Toll-like receptor 4 (TLR4), which recognizes DAMPs and then mediates downstream immune responses; upregulation of proinflammatory cytokines and chemokines, such as interleukin-1-beta (IL-1beta), IL-6, and tumor necrosis factor-alpha (TNF-alpha); the shift from healthy microglia and astrocytes to a primed or reactive state; disruption of the blood-brain barrier; cell death; and neurodegeneration.^20^,^49^–^55^ Although an acute inflammatory response is crucial for recovery and is initially beneficial to an organism after injury, the transition to chronic inflammation can eventually be detrimental.^56^ A state of chronic inflammation predisposes the system toward hyperreactivity, where a minor immune challenge can lead to an exaggerated response. This drives the organism further from homeostasis, leading to biological, cognitive, and behavioral dysfunction.^57^,^58^

It is not fully understood how the overlapping immune cascades interact after mTBI and subsequent alcohol consumption. In a recent study, mice that were fed an ethanol liquid diet after fluid percussion mTBI (which involves applying a brief fluid pressure pulse onto the brain through a hole in the skull) had elevated immune-related factors compared to control animals when treated with either alcohol or mTBI; levels of these factors were even higher in the mTBI + alcohol condition. This pattern held true for matrix metalloproteinases 2 and 9 (MMP2, MMP9); the key transcription factor nuclear factor-kappa B (NF-kappaB); the proinflammatory cytokines transforming growth factor beta-1 (TGFbeta-1), IL-1beta, TNF-alpha; the marker for microglia expression, ionized calcium-binding adapter molecule 1 (Iba1); and the marker for astrocyte expression, glial fibrillary acidic protein (GFAP).^59^ These results suggest that alcohol consumption and mTBI synergistically contribute to inflammatory outcomes and downstream immune signaling. Investigators also hypothesized that neuroinflammation itself may increase alcohol consumption through downstream effects, such as structural neurodegeneration and altered function in areas of the reward pathway (e.g., prefrontal cortex, ventral tegmental area, and the nucleus accumbens).^60^ No recent studies measured immunologic biomarkers in humans; however, one study suggested that anti-inflammatory diets (e.g., ketogenic, Mediterranean, or MIND diets) and supplementation with omega-3 polyunsaturated fatty acids and vitamin D may provide therapeutic benefits to individuals with mTBI, whereas alcohol consumption with its proinflammatory effects may be detrimental to recovery.^61^ This is likely because chronic inflammation and upregulated cytokine levels are associated with adverse biological and psychological outcomes after mTBI; thus, any additional sources of inflammation after injury are hypothesized to have a negative influence on overall health.^62^–^64^

The role of the microbiota in the gut and the gut–brain axis in alcohol use after mTBI is still underresearched but is a mechanism of interest and deserves further exploration. Interest in how the gut–brain axis is impacted by mTBI has only developed in the past couple of decades.^49^,^65^,^66^ Despite a fairly extensive literature on how alcohol affects the microbiome and gut–brain axis, it is not known whether changes in the microbiome directly drive alcohol use.^67^–^69^ Regardless, the gut–brain axis and its interaction with the immune system is an interesting area of emerging research. Some individuals with mTBI develop gastrointestinal symptoms, and AUD has long been associated with negative gastrointestinal outcomes.^70^,^71^ More recent work is beginning to uncover more specific changes in the gut–brain axis associated with mTBI and AUD, including altered mucosal permeability, changes in bacterial composition (dysbiosis) and movement (dysmotility), changes in metabolic pathways, and hormone dysregulation originating from the gut.^58^,^67^,^72^,^73^

#### Oxidative stress

Another mechanism relevant to the pathological progression of mTBI and AUD is oxidative and nitrosative stress. Oxidative and nitrosative stress occurs when there is an excess of free radicals, called reactive oxygen species (ROS) and reactive nitrogen species (RNS), respectively. When interacting with other molecules in the cells, these free radicals can then form toxic compounds. Free radicals are normally contained and neutralized by antioxidants, but an imbalance between radicals and antioxidants can lead to myriad biochemical disruptions, including lipid peroxidation, protein modification, mitochondrial dysfunction, impairment of the blood–brain barrier through loosening of vasculature and downregulation of tight junction proteins, induction of apoptosis and inflammation, and ultimately accelerated aging and neurodegeneration.^74^–^78^ It is hypothesized that oxidative stress occurs in TBI because the force of the impact or pressure wave induces mitochondrial and enzymatic activity, thus increasing ROS production. Alcohol use also promotes ROS formation: alcohol metabolism creates the byproducts H_2_O_2_ and acetaldehyde, and the interaction of H_2_O_2_ with copper or iron produces ROS.

Two recent studies quantified the expression of oxidative and nitrosative stress-inducing enzymes and markers of oxidative/nitrosative damage after mTBI and alcohol consumption in mice. The enzymes included nicotinamide adenine dinucleotide phosphate oxidase 1 (NOX1) and inducible nitric oxide synthase (iNOS), and the markers of damage included acrolein, 4-hydroxynonenal (4HNE), and 3-nitrotyrosine (3NT). Abdul-Muneer et al. observed elevated NOX1, iNOS, 4HNE, and 3NT in alcohol-only and mTBI-only groups, and further exacerbated levels in the mTBI + alcohol group.^59^ This suggests that oxidative stress may be a central mechanism that triggers other pathways of inflammation and neurodegeneration. Zhang et al. found elevated acrolein levels in multiple brain regions related to memory and anxiety in the mTBI + alcohol group only.^79^ The authors hypothesized that it was specifically the synergistic interaction of alcohol consumption after mTBI that damaged biochemical and cellular processes and induced the vulnerable state. Oxidative stress clearly is an important mechanism for AUD and mTBI, although more research is needed to interrogate the causality and progression of oxidative stress after mTBI and how it may contribute to increased alcohol consumption.

#### Neurodegeneration—Local cell death and loss of proteostasis

There is extensive literature on the role of AUD and TBI in development of dementia and neurodegeneration. Although results vary to some degree across clinical studies, and more standardized research is needed, both hazardous drinking and TBI are currently considered established risk factors for dementia and neurodegeneration, particularly if they occur during older age.^57^,^80^ Neurodegeneration can manifest as local cell death, which occurs acutely after injury, and as loss of proteostasis, which sometimes begins in the days to weeks after mTBI, but typically is not pathologically relevant until much later in life. Proteostasis is the homeostatic state of normal protein structure, expression, and functioning, and loss of proteostasis in amyloid-beta, tau, TAR DNA-binding protein 43 (TDP-43), alpha-synuclein, and other proteins can result in development of Alzheimer’s disease and related dementias.^81^ None of the clinical studies surveyed for this review found that alcohol consumption increased neurodegenerative markers (e.g., ISGylation, interferon beta, TDP-43, phosphorylated tau, or clinical cognitive assessment), whereas mTBI exposure did.^82^,^83^

Research conducted in a mouse model found that multiple measures of neurodegeneration (i.e., A-beta-42, phosphorylated tau, and markers of neuronal damage and apoptosis) were exacerbated by mTBI and alcohol together, compared with each alone or with control groups.^59^ In a drosophila model, researchers used a loss-of-function mutant in the *dTau* (*drosophila* Tau) gene, which is 44% identical and 66% similar to human Tau and models dysfunctional tau expression. mTBI and *dTau* loss of function each separately resulted in greater ethanol sensitivity, and there was an additive effect when mTBI was induced in the *dTau* loss-of-function mutant.^84^ Changes in ethanol sensitivity can result in changes in alcohol intake patterns, physiological response to alcohol, or both.^84^ Thus, TBI history and tau status likely play a role in alcohol use patterns. Another study investigated the role of a variant of the apolipoprotein E (*APOE4*) gene in mTBI and alcohol consumption (*APOE4* is the strongest known genetic risk factor for sporadic Alzheimer’s disease).^85^ The investigation was based on evidence that individuals with the *APOE4* gene who regularly consumed alcohol, especially females, were more vulnerable to cognitive impairment.^85^ The preclinical data indeed found an mTBI- and genotype-dependent effect on alcohol consumption, alcohol motivation, and risk-seeking behaviors. These studies demonstrate that genetic variants may help explain some of the heterogeneity in how mTBI and alcohol use influence the progression of neurodegeneration.

#### Neurodegeneration—Global loss of white and gray matter volume

More advanced stages of neurodegeneration and widespread cell death result in loss of white and gray matter volume. Gray matter is found on the surface of the brain and predominantly consists of cell bodies, whereas white matter is found in deeper brain structures and contains myelinated axons. The stereotypical temporal progression of tissue loss seen in normal aging differs from the pathological tissue loss seen after TBI, AUD, or in neurodegenerative diseases, such as Alzheimer’s disease, Parkinson’s disease, or frontotemporal dementia.^86^–^88^ Frontal and limbic structures are sensitive to degeneration after AUD or mTBI, which is in contrast to normal aging processes where limbic structures are typically spared.^55^

Measuring white and gray matter volume is readily done in humans but is far less common in mouse models due to their small size and lack of meaningful resolution. Magnetic resonance imaging (MRI) in a population of U.S. military service members and veterans was used to compare a calculated predicted brain age and chronological brain age.^86^ In males, deployment-related mTBI was associated with higher predicted brain age, whereas in females, a history of nondeployment mTBI was associated with older-than-expected brain age. Further analysis of the male participants showed that alcohol use was also associated with increased predicted brain age.

Diffusion-weighted MRI (dMRI) is a method that measures density of tissue, reflecting microstructural integrity and strength of axonal-dendritic connections. Rojczyk et al. performed dMRI in a cohort of veterans diagnosed with PTSD, substance use disorder, mTBI, or mood disorder. They found that veterans diagnosed with AUD or mTBI were more likely to engage in intimate partner violence, and those with higher psychological aggression had greater tissue density in the right amygdala-hippocampus complex.^89^ The study did not directly measure alterations in microstructural gray matter tissue integrity and timing of mTBI or alcohol use in this cohort of veterans; therefore, no definitive conclusions about causality can be made.

Both of the aforementioned studies, like a majority in the literature, measured gray and white matter volume *after* AUD or mTBI diagnosis. However, preexisting reductions in gray matter volume in regions related to decision-making, emotion, and reward processing could be a risk factor for hazardous alcohol consumption or mTBI.^90^ This interpretation may be harder to confirm in clinical studies due to limitations in establishing causality but could improve understanding about how tissue volume influences behavior.

#### Neuroendocrine alterations

The neuroendocrine system is vast and complex and is influenced by many lifestyle and environmental factors. It is responsible for the regulation of homeostatic and adaptive physiological processes and functions through hormone secretion and signaling mainly from the hypothalamus, pituitary gland, and adrenal glands. This hypothalamic-pituitary-adrenal axis is responsible for the stress response after physical or psychological challenges and trauma. One of the primary hormones involved in the stress response is cortisol (known as corticosterone in rodent models). No papers surveyed for this review specifically measured cortisol or corticosterone, but other preclinical, clinical, and review papers found that cortisol/corticosterone dynamics are complicated. The hormones often show elevated levels in response to acute stress but blunted response after chronic stress; there is also a strong circadian influence.^91^–^95^

A recent study quantified both hormones from the pituitary gland (i.e., follicle-stimulating hormone, luteinizing hormone, adrenocorticotropic hormone, and thyroid-stimulating hormone) and hormones from pituitary target organs (i.e., testosterone, cortisol, and free thyroxine) after mTBI and alcohol consumption to determine pituitary function.^96^ Twelve months after mTBI, 16% of individuals had developed pituitary deficiency. The affected population was more likely to be younger and female, but there were no associations between pituitary deficiency and injury modality or complications. Individuals with mTBI who consumed alcohol at low-risk or heavy-drinking levels were less likely to develop pituitary dysfunction, whereas those with mTBI who did not consume alcohol regularly had increased risk.

Corticotropin-releasing factor (CRF) is a neuropeptide that is released from the hypothalamus and then acts upon the pituitary; it is often studied in relation to trauma and/or alcohol use. In a recent study, lower levels of CRF were able to distinguish individuals with PTSD from those with PTSD and mTBI, which often occur together.^97^ The PTSD group was then split into individuals with AUD and those without. The level of CRF did not differ significantly between these groups, although the PTSD + AUD group had less variability in their CRF levels than the PTSD without AUD group. Taken together, this suggests that although dysregulation of CRF is not specific to mTBI, the correlation with PTSD means that it may be more relevant for the psychological aspect of trauma and could be connected to alcohol use in that way.

The dynorphin/kappa opioid receptor (KOR) system plays an important role in the neuroendocrine system and stress response through its expression across the HPA axis and interactions with CRF.^98^ The dynorphin/KOR system independently plays roles in mTBI and alcohol use, and is hypothesized to be a potential link between the two.^99^ Although the entire signaling cascade is more complicated than can be fully appreciated here,^100^ it is generally thought that dynorphin and KOR activity increases after mTBI, leading to a dysphoric state.^101^ Dynorphin/KOR activation causes decreased tonic dopamine levels, thereby disrupting reward pathways and behaviors, promoting negative affect/anxiety-like behaviors, and enhancing disinhibition.^102^–^106^ The resulting state mediated by the dynorphin/KOR system affects neurotransmitters, neuropeptides, excitation-inhibition balance, and inflammation, which is hypothesized to “neurochemically prime” the brain toward hazardous alcohol and substance use behaviors.^60^

Brain-derived neurotrophic factor (BDNF) also helps to regulate the neuroendocrine system and the stress response. BDNF has neuroprotective properties and is involved in neuroplasticity, neurogenesis, synaptogenesis, and cell survival. BDNF signaling is affected by stress exposure and is implicated in many mood disorders in addition to AUD and mTBI.^107^,^108^ BDNF has been linked to mTBI because of its role in the repair and regeneration of neurons, and different genetic variations of BDNF may influence outcomes after mTBI.^109^–^111^ There also has been interest in how alcohol consumption affects BDNF and its role in neuroplasticity and how BDNF signaling may affect alcohol consumption.^112^ Based on previous research, it can be hypothesized that if BDNF normally plays a role in regulating the moderation of alcohol consumption, then an mTBI exposure that disrupts BDNF signaling may lead to escalated alcohol intake or preference.^113^ A recent paper found that BDNF levels predicted development of PTSD after mTBI only in individuals who had consumed any level of alcohol in the past year, further implicating BDNF in the synergistic interactions of mTBI and alcohol use.^114^

Another component of the neuroendocrine system is the endocannabinoid (eCB) system, which regulates many functions in the body and plays a role in homeostasis. Downstream mechanisms affected by eCB signaling include neuroinflammatory effects, blood–brain barrier permeability, cerebrovascular modulation, glutamate excitotoxicity, and neuroplasticity. Both alcohol and mTBI dysregulate eCB functioning.^115^–^117^ The eCB system is a potential target for new therapies (for mTBI, AUD, and number of other conditions) because it exerts neuroprotective effects in response to detrimental stimuli.^118^ The drug JZL184 (4-nitrophenyl 4-[bis(2H-1,3-benzodioxol-5-yl) (hydroxy)methyl]piperidine-1-carboxylate) inhibits degradation of eCBs, effectively prolonging their activity and function. Treatment of fluid percussion mTBI with JZL184 improved neurological and behavioral outcomes, blood–brain barrier integrity, and glial activation and decreased alcohol motivation in male rats.^119^–^121^ A follow-up study found that a repetitive closed-head weight drop model of mTBI (which involves dropping a small weight or rod from above the skull to produce a focal cerebral contusion as well as diffuse injury mechanisms) induced higher alcohol consumption in female but not male adult rats, and increased anxiety in male but not female adult rats.^122^ JZL184 did not affect alcohol behavior in either sex; however, in contrast to the previous research, it increased anxiety-like behavior in male rats at 1 week post mTBI. The researchers hypothesized this could be due to the dose level or specific timing of drug administration, supporting the idea that eCB signaling is dynamic and can have differential effects.

#### Dopamine signaling

Many problematic behavioral changes that are associated with mTBI and/or AUD concern behaviors that are typically associated with dopamine signaling. Biochemically, alcohol has rewarding properties that directly affect dopamine signaling and are implicated in motivated behavior.^123^ The literature about alterations in neurotransmitter signaling, especially dopamine signaling, that occur after mTBI and influence alcohol drinking behaviors is primarily based on preclinical models (for specific studies, see the review articles by Weil et al.,^55^ Merkel et al.,^60^ and Gallant and Good^124^). It is difficult to study dopamine dynamics in humans. Nevertheless, people with TBI are sometimes prescribed drugs that increase dopamine signaling (e.g., methylphenidate, amantadine, bromocriptine) due to their effectiveness in treating behavioral symptoms, including working memory, attention, mental fatigue, cognitive processing speed, social functioning, and depression.^125^,^126^

It is thought that corticostriatal dopamine levels increase acutely after mTBI, resulting in a hyperdopaminergic state. At some point between 24 hours and 2 weeks after injury, corticostriatal regions then transition to a hypodopaminergic state.^1^,^60^,^124^ In contrast, evoked dopamine responses in the nucleus accumbens are increased at both 4 to 7 days and 30 days after (blast) mTBI.^127^ There is also a bidirectional relationship between dopamine signaling and inflammation as the release of dopamine may increase inflammation but the availability of dopamine precursors is reduced after an inflammatory response.^55^ Further, both the primary phase (impact forces) and the secondary phase (oxidative stress) of TBI can damage dopaminergic neurons. Dopaminergic white-matter tracts, such as the mesocorticolimbic pathway, are particularly affected by the stretching and shearing that occurs during some injury modalities.^128^,^129^ One recently published study found that male, but not female, mice showed reduced numbers of cells expressing the dopamine receptor D2 in various areas of the hippocampus (i.e., dentate gyrus, lacunosum moleculare, and CA3 areas) 4 weeks after fluid percussion TBI.^130^ This confirms previous findings of a chronic hypodopaminergic state after TBI, and although the hippocampus is not part of the corticostriatal or mesolimbic dopamine pathways, it has been strongly implicated in memory and is one of the first areas affected by neurodegeneration.

Alcohol directly acts on the mesolimbic dopamine system, also known as the reward pathway. Alcohol consumption causes a release of dopamine in the nucleus accumbens, and other alcohol-related behaviors—such as alcohol seeking, craving, and withdrawal—are hypothesized to have biological substrates in the mesolimbic pathway and to affect dopamine signaling (for reviews of alcohol’s interactions with the dopamine system, see Siciliano et al. 2018,^123^ Söderpalm and Ericson 2024,^131^ and Wise and Jordan 2021^132^). Because disruptions in dopamine signaling after mTBI have an effect on reward circuitry, alcohol-related behaviors likely would also be affected. Moreover, aberrant dopamine transmission in brain regions, such as the prefrontal cortex affect cognition and decision-making, which can also impact hazardous drinking.^133^

### Behavioral Impairments Connecting TBI and AUD

The literature clearly indicates that behavioral changes after TBI significantly contribute to decreased quality of life post-injury.^16^,^31^,^134^–^137^ Physiological changes after injury are undoubtedly important and contribute to disease progression, but underlying biological changes can go unnoticed in daily life. Impairments in cognition, executive functioning, and mood or alterations in decision-making, irritability, and impulsivity may be more apparently detrimental and easier for others to notice. This section reviews the behavioral changes that are common after TBI, the relationship with hazardous alcohol consumption, and how they may lead to the development of AUD.

#### Cognitive and reward processing deficits

Cognitive deficits after TBI are common and include problems with attention, memory, and executive function (e.g., planning, organization, decision-making, and goal-oriented behaviors).^138^–^140^ Behavioral symptoms sometimes appear immediately and persist chronically; in other cases, they may not develop until years after the initial injury. Dopamine signaling in brain regions such as the prefrontal cortex, striatum, hippocampus, thalamus, and others plays an important role in cognition and reward processing.^141^ Many hypothetical models attempting to explain a scientific basis for addiction are based on impairments in decision-making, goal-oriented behavior, and reward-processing (e.g., incentive salience, incentive sensitization, reward-deficiency, and ambivalence).^142^–^147^ These behaviors sometimes develop after mTBI, and as described below, they may play a role in driving negative outcomes and hazardous drinking after injury.

Following TBI, individuals may give higher reward value to alcohol than they did before the injury.^124^ On the one hand, this change may have a biological basis in disrupted reward circuitry in the brain or biochemical changes that affect the rewarding properties of alcohol. On the other hand, it may in part be due to psychosocial factors, such as desire for social cohesion, maladaptive coping mechanisms, and alcohol’s ability to reduce anxiety and negative mood (which will be discussed further in the next section). Individuals with TBI may also have issues with anticipating and weighing future consequences, which can lead to hazardous drinking behaviors.^55^ Improper appraisal of the potential benefits and/or costs of a decision can lead to a suboptimal decision, which is seen in individuals with TBI during clinical tasks.^148^ Impairments in executive functioning may also increase the prevalence or severity of alcohol use by impacting efficacy of therapeutic interventions. For example, difficulty with attention, planning, and organization may interfere with the ability to start or continue treatment. Furthermore, aspects of treatment that utilize self-reflection or critical thinking may be more difficult after TBI, such that individuals with TBI may not respond to typical treatment strategies and require other interventions.^1^

A study of nearly 200,000 veterans found that the greatest risk factor for receiving a diagnosis of dementia or mild cognitive impairment was history of mild or moderate TBI, and that either a history of high alcohol use *or* current high alcohol use was also associated with cognitive deficits when controlling for previous TBI.^34^ In this study, high alcohol use was defined as a score of 6 or higher on the AUDIT-C. A smaller study of veterans with substance use disorder did not find an association between TBI history and cognitive deficits but did find that higher impulsivity and lower cognitive performance were correlated with higher health care costs within the VA Hospital system.^149^ The study did not report the specific classification of TBI for each participant, but overall the study population was described as reporting a low severity of TBI. These results indicate that individuals experiencing behavioral impairments may have worse outcomes and also suggest an opportunity for a point of intervention that may improve health care outcomes and costs.

In a sample of civilians in Uganda, 56% of individuals with TBI reported cognitive deficits. Severity of TBI (mild/moderate/severe) was not reported; however, loss of consciousness was associated with a higher risk of cognitive deficits, indicating some relationship between TBI severity and cognitive impairment.^150^ Alcohol use appeared to be protective against cognitive impairment in this study. Several other studies also have proposed that light alcohol consumption may have no effect or potentially even protective effects on cognitive function, although results are mixed.^151^–^153^ However, there are no recommended safe drinking levels, and many health organizations recommend limiting consumption due to the negative effects of chronic heavy consumption, binge drinking, and AUD.^154^,^155^

#### Impulsivity, disinhibition, and affect

Another common behavioral phenotype that is observed after mTBI is impulsivity, which is related to the personality trait of disinhibition.^127^,^156^ Impulsivity refers to acting without thinking and without regard for consequences (i.e., it represents a specific behavioral expression), whereas disinhibition is defined as a lack of control over unwanted behaviors and poor self-regulation (i.e., it represents a stable personality trait characterized by aggressive, irritable, and risk-taking behaviors in addition to impulsivity).^157^ Disinhibition and impulsivity are predictive of AUD even in non-mTBI populations, and it has been hypothesized that aberrant dopaminergic signaling (as seen in mTBI) plays a role in how disinhibitory behaviors influence the onset and development of AUD.^55^,^124^,^158^,^159^ Two recent clinical studies of civilian populations indeed found that those with a history of mTBI had the highest endorsement of impulsivity, irritability, anger expression, and antisocial behavior; however, only one of the studies observed higher rates of AUD.^137^ Alcohol use after mTBI in civilian populations tends to be more varied than in veterans or contact sport athletes, and the study that did not find a relationship only consisted of 368 participants total.^160^

Affect and mood disorders may also contribute to increased alcohol consumption after mTBI. Decreased mood and increased anxiety are seen in approximately 25% to 50% of individuals who have experienced TBI, and mood disorders are independently associated with increased rates of alcohol use.^134^,^161^ One hypothesis is that alcohol can be used as a maladaptive coping strategy to avoid negative emotions, thoughts, and feelings, all of which may increase after mTBI.^162^ Further, individuals who use alcohol to avoid negative states tend to show more hazardous and binge-like consumption patterns; conversely, those who drink for enhancement of positive states and social reasons tend to consume alcohol more moderately.^55^,^163^ Although an in-depth discussion of PTSD is outside the scope of this article, it should be mentioned that mTBI and PTSD are highly comorbid, as are PTSD and AUD. Research on the mechanisms and behaviors specifically relevant to PTSD has been done, although additional studies can help identify effective treatments.^2^,^11^,^54^,^164^

## Discussion

### Conclusions of the Review

Decades of research have been conducted on either mTBI or alcohol use alone. However, preclinical and clinical research only started to focus on the onset of AUD and the negative outcomes associated with hazardous drinking after mTBI in the past 15 years, and particularly in the last decade. Further research in this area that builds upon the existing literature may help to better understand the progression of symptoms and mechanistic changes that occur with alcohol use after mTBI, preexisting factors that influence this progression, and possible interventions that may prevent hazardous drinking patterns and negative chronic outcomes.

Perhaps the most striking conclusion from this review is the heterogeneity in injuries and outcomes among individuals who experienced mTBI. Many individuals report no apparent long-lasting symptoms after injury, whereas others experience persistent symptoms for months to years after mTBI. In yet another subset, changes in behavioral and biological systems and functions go unnoticed until they manifest years after mTBI.

The stereotypical acute injury mechanisms after mTBI include oxidative stress, cell death, and an inflammatory response. Over time, this progresses into some combination of chronic inflammation, neuroendocrine alterations that affect a wide array of systems across the body, changes in dopamine signaling, and global gray- and white-matter loss. These biological changes manifest in various cognitive and neuropsychiatric behavioral deficits after injury, and people with mTBI have a higher risk for mild cognitive impairment or dementia. Although most studies—this review included—discuss all of these mechanisms separately, physiological and behavioral changes after mTBI occur in parallel as well as synergistically. These impairments not only have the potential to contribute to increased hazardous alcohol consumption, but also to interact with the cascade of effects resulting from alcohol consumption. The synergistic interaction of biological mechanisms means that people with mTBI who consume alcohol may experience more, and more severe, impairments than their uninjured peers who use alcohol. Military populations, contact-sport athletes, and adolescents are especially vulnerable to the impacts of mTBI and often see worse outcomes. Potential explanations include environmental factors that may be more prevalent for service members than the civilian population, the repetitive nature of injuries in athletes, and the continued development of neurological and peripheral systems in adolescence.

### Limitations

This review is limited in several ways. The clinical papers identified include a wide range of populations, age ranges, time frames after injury, and injury modalities that affect outcomes. For example, blast and rotational injuries likely result in different downstream mechanistic changes than do penetrating or blunt force injuries. While the variety of papers reflect the heterogeneity of how mTBIs and hazardous alcohol use manifest in individuals across many populations and scenarios, the broad nature of the search prevented in-depth discussion of all relevant populations and mechanistic impairments. Another important consideration is that across populations, TBI exposures are often underreported, especially mTBI. For example, people may not always realize that they received an mTBI; in other cases, accurately reporting symptoms may prevent participation in sports or military activities. Numerous lifestyle factors that can significantly impact outcomes after TBI also were not considered in every study, including sleep, physical activity, diet, social environment, environmental pollutants or toxins, family history, or genetic factors. In clinical studies, details regarding the exact amount and timing of alcohol consumption or the amount of time since injury may not be precise, which could also affect the conclusions that are drawn. Finally, a significant number of individuals who experience TBI are also diagnosed with PTSD. It is difficult to fully understand the separate contributions of TBI and PTSD to the resulting biological and behavioral symptoms (for further discussion of this topic, see Hendrickson et al.^11^). This review did not separate individuals with only TBI from those with TBI and PTSD unless it was specified in a specific study.

The preclinical literature also had some pressing limitations to be addressed in future research. Female subjects were often not included, especially in research involving blast models of TBI. Additionally, there was a paucity of research on how mTBI exposure interacted with alcohol use in older individuals, even though older adults are experiencing TBIs at a higher rate than any other age group and exhibit continually increasing rates of binge drinking.^165^,^166^ Perhaps the most important limitation was that there were limited preclinical studies that included mTBI exposure and alcohol access in the same model. A significant proportion of knowledge about mTBI and alcohol interactions has come from evidence obtained in studies of the individual conditions where the outcomes seen after mTBI overlapped with mechanisms seen with hazardous alcohol drinking.

### Future Directions

To date, there are fewer than 10 published studies in which preclinical subjects were exposed to mTBI and then given access to alcohol in the same cohesive experiment.^35^,^59^,^79^,^82^,^84^,^85^,^122^ Future research would benefit from this experimental design because it has translational relevance and could improve understanding of the synergistic interactions between mTBI and alcohol more effectively than studying the two conditions in separate subjects and models.

Greater knowledge about the underlying mechanisms that occur after mTBI and how they influence the onset of AUD could improve how individuals with mTBI are treated across many populations. Basic and translational research would allow for advances in therapies and interventions to improve outcomes at multiple stages of injury progression. Additionally, studies that investigate the interactions across multiple mechanisms could move the field forward. For example, research could examine how oxidative stress leads to gray-matter loss, how inflammation exacerbates aberrant neuroendocrine signaling and hormone release, how changes in the microbiome affect behavior, and similar questions. Future research that looks holistically at both central and peripheral systems and that includes historically understudied populations could make tangible progress in understanding how mTBI drives development of AUD.

**KEY TAKEAWAYS**

- Mild traumatic brain injury (mTBI) can result in behavioral and physiological symptoms that develop acutely and persist for years after the initial injury, including increased hazardous alcohol use.
- Neuroinflammation, neurodegeneration, neuroendocrine alterations, aberrant dopamine signaling, and deficits in executive function and reward-processing may contribute to the mechanisms by which TBI and hazardous alcohol use synergistically lead to negative chronic outcomes.
- Future holistic research looking at biological and behavioral changes that includes females and older subjects could help clarify the relationship between TBI and alcohol.
